# Preoperative ultrasound-guided carbon nanoparticles localization for metastatic lymph nodes in papillary thyroid carcinoma during reoperation: A retrospective cohort study

**DOI:** 10.1097/MD.0000000000006285

**Published:** 2017-03-10

**Authors:** Wan-jun Zhao, Han Luo, Yi-mei Zhou, Ze-hui Gou, Bin Wang, Jing-qiang Zhu

**Affiliations:** a Department of Thyroid & Parathyroid Surgery; b Department of Ultrasound, West China Hospital; c West China School of Stomotology, Sichuan University, Chengdu, PR China.

**Keywords:** localization, reoperation of papillary thyroid carcinoma, ultrasound-guided carbon nanoparticles dyeing

## Abstract

Due to the damaged anatomical structure and a large amount of fibrous and scar tissues in the surgical field, reoperation of papillary thyroid carcinoma is difficult. This study introduces a new method of locating metastatic lymph nodes during reoperation and evaluates the effectiveness and safety of the preoperative ultrasound-guided carbon nanoparticles (CNs) localization. This retrospective cohort study enrolled 52 patients who were diagnosed with lymph node metastasis by histopathology and underwent reoperation from October 2015 to February 2016. The modified radical neck dissection or selective neck node dissection was performed. A total of 26 patients underwent preoperative ultrasound-guided CNs injection, and other 26 patients did not. Tolerance, the result of injection, the number of resected metastatic lymph nodes, and postoperative complications were recorded and analyzed. In CNs group, 102 suspicious nonpalpable lesions in 26 patients were injected with CNs, and 99 of the 102 lesions were successfully identified by surgeon in the reoperation. The positive rate of resected lymph nodes in total, in the central compartment, and in the lateral compartment were 31.6%, 31.2%, and 32.8%, respectively, which was significantly higher than that in the control group (*P* < 0.001, *P* < 0.001, and *P* = 0.041). In addition, the positive rates of levels III, IV, and V in the CNs group were 35.6%, 21.9%, and 30.5%, respectively, which was significantly higher than that in the control group (*P* < 0.001, *P* = 0.005, and *P* = 0.01). In additional, in the CNs group, the rate of temporary hypoparathyroidism was significantly lower compared with the control group (0% vs 26.9%, *P* = 0.021). Preoperative ultrasound-guided CNs injection is a safe and effective method for localization of the metastatic lymph nodes during reoperation.

## 1 Introduction

Papillary thyroid carcinoma (PTC) is a common malignant tumor, which has an increased incidence rate globally. After the confirmed diagnosis of PTC, the conventional treatment is surgical excision. But it has a high rate of postoperative lymph node recurrence or metastasis, up to 5% to 40%, because of its biological characteristics that it can easily invade the surrounding lymph nodes.^[1]^ Besides, with the development of long-term follow-up for thyroid cancer patients, ultrasound, and serum thyroglobulin (HTG) monitoring as the common detection index, the diagnosis rate of PTC recurrence is significantly increasing.^[2,3]^ Treatment with I^131^ radiotherapy alone was effective for some patients with recurrent and metastatic lesions. Nevertheless, up to 30% of PTC is not sensitive to I^131^, which sets obvious limitation on I^131^ radiation therapy.^[4]^ American Thyroid Association guidelines indicated that surgery was the optimal treatment for the recurrence of PTC, which has given perfect performance.^[5]^ However, the damaged anatomical structure and a large amount of fibrous and scar tissues in the surgical field of reoperation make it difficult for the surgeons to find the suspicious lymph nodes and prolong the operative time. Moreover, the incidence rate of some complications, such as permanent hypoparathyroidism (HPT) and recurrent laryngeal nerve injury, is increased simultaneously. Therefore, we need a method to locate suspicious lymph nodes to avoid more injury.

Carbon nanoparticles (CNs), as a new lymphatic tracer with the average diameter of 20 nm, can directly enter the lymphatic capillaries but not the blood capillaries,^[7]^ because its size is smaller than the gap between the lymphatic endothelial cells but larger than the gap of capillary endothelium. CNs have been used for dyeing breast cancer, gastric cancer, and rectal cancer for more than 5 years.^[6–11]^ In recent years, they are commonly used as intraoperative localization for thyroid cancer, which would bring extraoperative procedure.^[12,13]^ Preoperative ultrasound-guided CNs localization will improve this disadvantage. Therefore, we conducted a retrospective cohort study to evaluate the effectiveness and safety of reoperative ultrasound-guided CNs localization.

## 2 Materials and methods

### 2.1 Patients’ selection

From October 2015 to February 2016, this retrospective cohort study enrolled 52 patients with diagnosed lymph node metastasis by histopathology. They underwent reoperation in the department of Thyroid & Parathyroid surgery of West China Hospital of Sichuan University. This study was approved by the Ethics Committee of West China Hospital of Sichuan University, and informed consents were written by patients agreeing to participate. Patients who met the following criteria were recruited: had PTC once, had already accepted thyroid surgery, were confirmed as lymph node recurrence by fine-needle aspiration biopsy (FNAB), all the suspicious lymph nodes were nonpalpable, and agreed to accept reoperation. Conversely, we excluded patients who had medullary, anaplastic, or follicular thyroid carcinoma; who did not undergo FNAB; who voluntarily gave up reoperation; and who had follow-up less than 6 months. Basic data that were retrospectively collected such as age, gender, type of first surgery, Tumor Node Metastasis (TNM) classification, I^131^ therapy, and radiotherapy are summarized in Table 1. Before reoperation, as patients’ informed preferences, ultrasound-guided CNs dyeing was conducted in 26 patients in CNs group, while 26 patients in control group did not undergo any dyeing procedure.

**Table 1:**
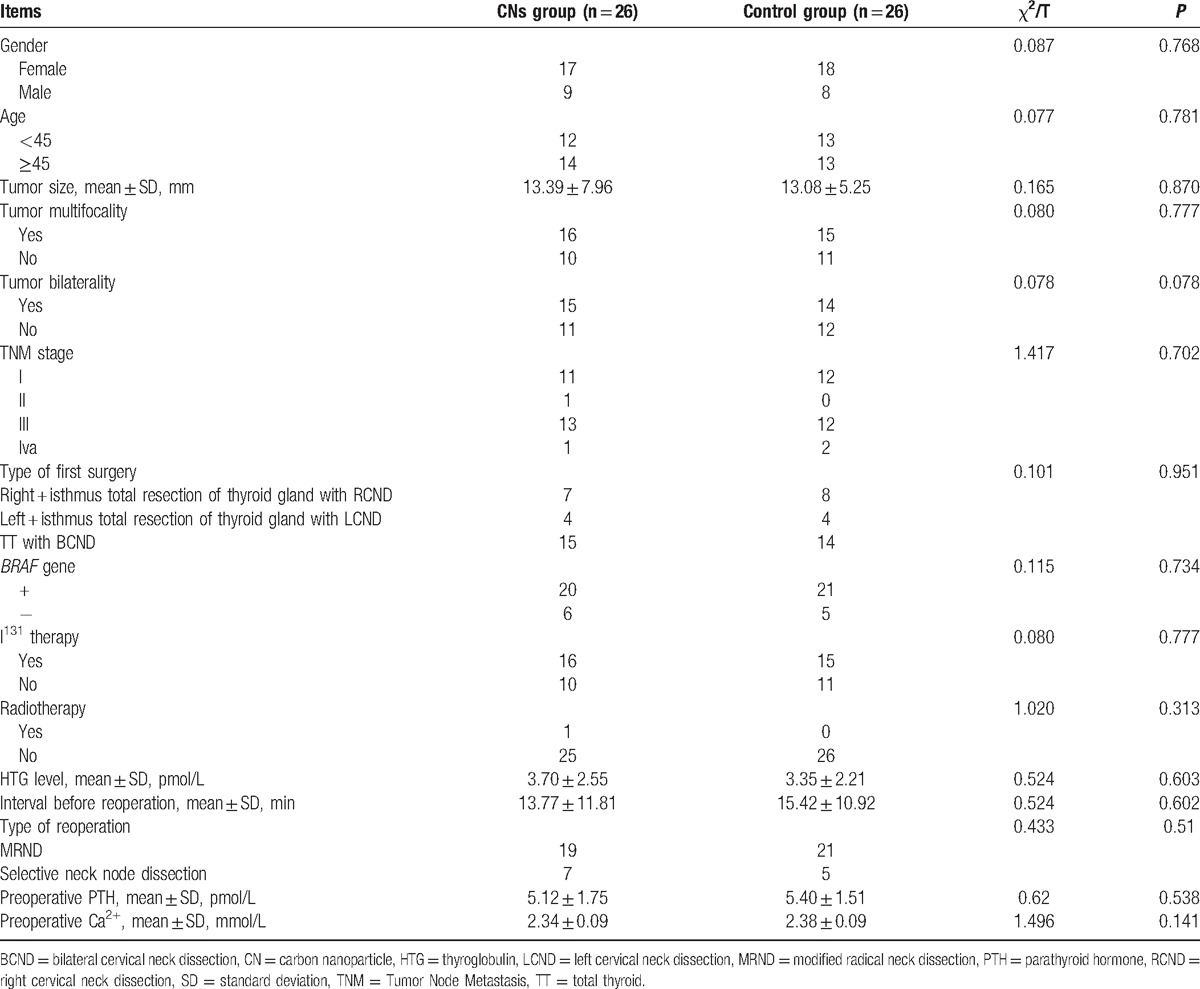
Comparison of clinical characteristics of patients who underwent carbon nanoparticles injection or not.

### 2.2 Ultrasound-guided CNs dyeing procedure

**Figure 1:**
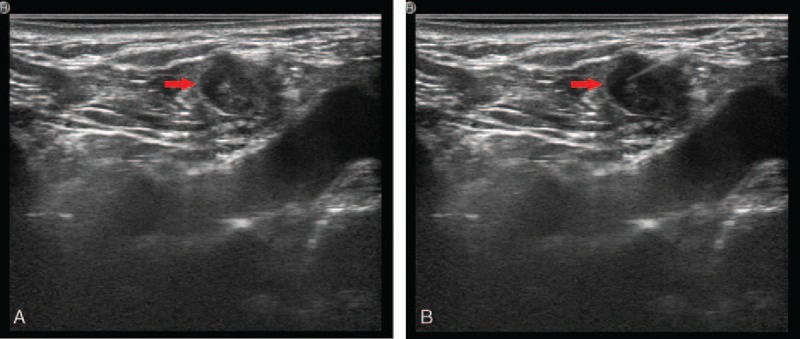
(A) Ultrasound image of a suspicious lymph node. The red arrow shows the site of the suspected lymph node under the ultrasound guiding. (B) Carbon nanoparticles injection. The red arrow shows the site of the suspected lymph node under the ultrasound guiding.

All patients received ultrasound workup before reoperation to evaluate. Suspected lymph nodes on ultrasound were shown as ratio of long/short diameter <2, hyperechogenicity, calcification, and heterogenous inner structure.^[14]^ We choose CNs suspension (kanalin; Lummy Pharmaceutical, Chongqing, China) as dyeing materials of localization in 1 to 14 days before reoperation. Under the ultrasound guiding, CNs were injected into the suspicious lesions with 1-mL syringe by an experienced radiologist (ZG). The radiologist would choose a safe and short way to avoid puncturing into vessels and nerves. A volume of 0.05 to 0.1 mL of dyeing materials was injected into suspicious lesions based on the size of lesions. At least 1 hour of observation after injection was needed, so that timely disposal could be arranged once patients felt unwell. All information about dyeing procedure was recorded such as location, amount, and dose and sent to the main surgeon (Fig. 1A and B).

### 2.3 Surgical procedure

All patients received general anesthesia with endotracheal intubation and the same surgical procedure. According to ultrasound imaging, we chose different surgical site. If suspicious metastatic lesions were concentrated in the central compartment (level VI) or dispersed, an arch incision along with original incision would be made or extended. Then the modified radical neck dissection (MRND) was performed. If they are only located in lateral compartment (e.g., level IIB or level V), we would make a 2 to 3-cm incision on these locations to avoid unnecessary injuries and perform selective neck node dissection. Not only the suspicious lesions, but also other lesions that ultrasound had not recognized before in the surgical field were resected.

### 2.4 Monitoring indicators

During dyeing procedure, we recorded the injection site and the number of injected lesions. Besides, we also evaluated the injection complications, such as intolerable pain, black marking at the skin puncture site, hematoma, and so on.

For reoperation, we compared the operative time, the number of resected and stained lymph nodes, positive rate of resected lesions (the number of metastatic lymph nodes/the number of total resected lymph nodes), and postoperative complications of patients with and without CNs dyeing.

We identified the function of recurrent laryngeal nerve by laryngoscopy (Timcke KS-4200/S, Hamburg, Germany) and analysis of voice. The evaluation of the tone was mainly through a portable tone recorder (TCD-D3 serial, number Sony 901802, Japan; Sony ECM-MS907 microphone, Japan) and multidimensional sound system (MDVP; Elemetrics Kay, Brook Pine, NJ). In addition, all the patients should undergo laryngoscopy 1 day before reoperation. Patients who have postoperative hoarseness found by pronunciation performance should undergo another laryngoscopy to confirm whether they have vocal cord paralysis. HPT was defined as serum parathyroid hormone (PTH) below the normal range (1.60–6.90 pmol/mL), regardless of hypocalcemic symptoms. Permanent HPT was defined as serum PTH less than the normal range over 6 months.^[15]^

### 2.5 Statistical analysis

Statistical analysis was performed by SPSS version 19.0 (SPSS Inc, Chicago, IL). All data are expressed as mean ± standard deviation. Chi-square test and *t* test were used to analyze variables. For all tests, *P* value smaller than 0.05 was considered statistically significant.

## 3 Results

This retrospective cohort study recruited 52 patients (35 females and 17 males) with PTC who underwent the reoperation. The mean age was 44.60 ± 8.34 years. Table 1 compares the basic characteristics of the patients with or without CNs injection before reoperation. There was no significant difference between the 2 groups in gender, age, primary tumor size, multifocality, bilaterality, TNM stage type of first surgery, *BRAF* gene, I^131^ therapy, radiotherapy, HTG level, interval before reoperation, type of reoperation, preoperative PTH, and Ca^2+^ (*P* > 0.05).

### 3.1 Tolerance and results of CNs injection

We observed that 26 patients with CNs did not have insufferable pain or bleeding during the injection procedure. They felt that it was just like the ultrasound-guided FNAB, which all of them had previously experienced. Besides, any clinical visible inflammatory reaction or hematoma was not found. During the 6 months of follow-up period, there was no side effect due to CNs injection in the CNs group. But it was noteworthy that 1 patient (3.8%) had skin marking at the puncture site after injection.

Preoperative ultrasonography showed that there were 102 suspicious nonpalpable lesions in 26 patients with CNs. All the lesions were injected with CNs, and 99 of 102 lesions were successfully identified by surgeon in the reoperation. The number of lesions injected at levels II, III, IV, V, and VI were 9 (8.8%), 34 (33.3%), 17 (16.7%), 14 (13.7%), and 28 (27.5%), respectively. In addition, there were 23 lesions near the major neck vessels successfully injected. For the 3 (2.9%) failed lesions, the dye areas were found in the surrounding tissues about 2 to 3 cm near the lesions, and all 3 lesions were resected successfully. The postoperative pathological results showed that 78 of 102 lesions (76.4%) were metastatic lymph nodes.

**Figure 2:**
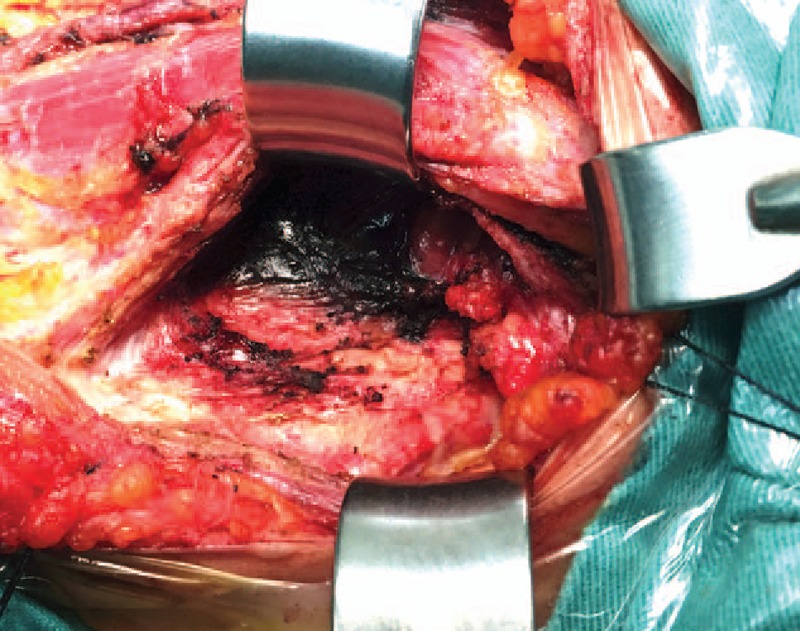
Lymph node marked with carbon nanoparticles during the reoperation.

**Figure 3:**
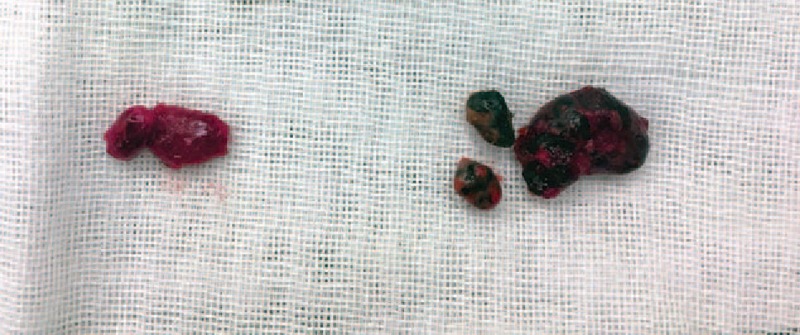
Comparison of the resected lymph nodes with or without carbon nanoparticles dyeing. On the left are the resected lymph nodes without carbon nanoparticles dyeing. On the right are the resected lymph nodes with carbon nanoparticles dyeing.

In the CNs group, we resected 528 lymph nodes. Except the preoperative suspicious lesions, there were 426 resected lesions without CNs injection. A total of 234 of 426 lesions (54.9%) were also dyed, and 89 of 426 lesions (20.9%) were metastatic lymph nodes defined by postoperative pathology test (Figs. 2 and 3).

### 3.2 Comparison of the resection of metastatic lymph nodes in reoperation

The number and ratio of resected and metastatic lymph nodes in reoperation are listed in Table 2. The positive rate of resected lymph nodes in total, in the central compartment and in the lateral compartment, were 31.60%, 31.20%, and 32.80% in the CNs group and comparatively 18.50%, 15.90%, and 23.90% in the control group. The positive rate of resected lymph nodes in total, in the central and lateral compartments, had significant difference between the 2 groups (*P* < 0.001, *P* < 0.001, and *P* = 0.041). With regard to the positive rate of resected lymph nodes in the lateral compartment neck levels (II, III, IV, and V) in the 2 groups, the positive rate of level II was similar. However, we found that the positive rate of levels III, IV, and V in the CNs group were significantly higher than that in the control group (*P* < 0.001, *P* = 0.005, and *P* = 0.01).

**Table 2:**
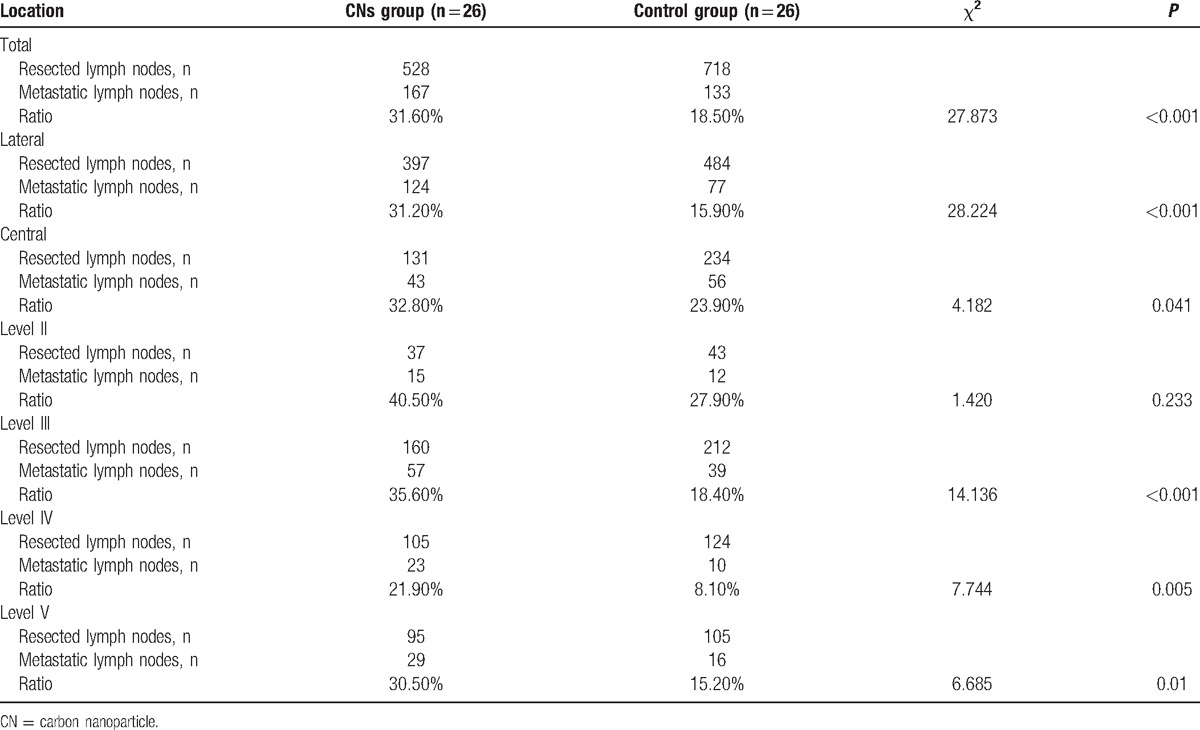
Comparison of resected and metastatic lymph nodes with and without carbon nanoparticles injection.

### 3.3 Postoperative complications

In Table 3, we can know that the reoperation time in CNs group was significantly shorter, compared with the control group (*P* = 0.001). The follow-up period was more than 6 months for 52 patients. There was no occurrence of vocal cord paralysis in CNs group, but 2 patients in control group had transient vocal cord paralysis after reoperation. The occurrence rate of vocal cord paralysis in control group was 7.6%, which indicates no significance between the 2 groups (*P* = 0.471). The convalescence of the 2 patients was 4 and 7 weeks.

**Table 3:**
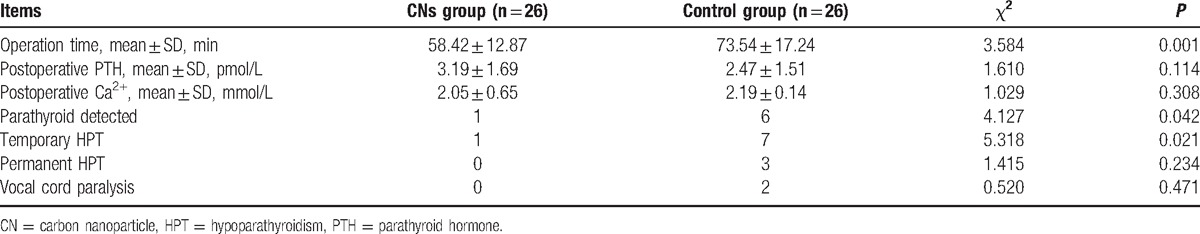
Comparison of surgical outcomes for patients with and without carbon nanoparticles injection.

In addition, the rate of patients with parathyroid detected by postoperative pathological examination was 3.8% (1/26) and 26.9% (6/26) in CNs and control groups, respectively. There was significant statistical difference between the 2 groups (*P* = 0.042). The rate of temporary and permanent HPT in CNs group was 3.8% (1/26) and 0% (0/26), respectively. The rate of temporary and permanent HPT in control group was 26.9% (7/26) and 11.5% (3/26), respectively. In the CNs group, the rates of temporary HPT were significantly lower compared with that of the control group (*P* = 0.021). But the difference between the 2 groups did not reach statistical significance for the permanent HPT, the postoperative PTH, and Ca^2+^ (*P* = 0.234, 0.114, and 0.308). Moreover, so far, our follow-up showed that there was no recurrence in the 2 groups.

## 4 Discussion and conclusion

For the PTC, the follow-up with cervical ultrasonography and monitoring of serum HTG level allow the early detection of smaller nonpalpable recurrence or metastasis. In addition, reoperation is the main curative treatment for the recurrence of PTC. However, reoperation is a technical challenge. In our experience, the occurrence risk of complication in reoperation is 5 to 10 times higher than that in initial operation.^[16–19]^ Moreover, because of the fibrosis tissue formation and the anatomic damage in the surgical field, the effectiveness of the detection of metastatic lymph nodes would be lower, especially when lymph nodes are surrounded by the fibrosis tissues. Therefore, to identify the metastatic lymph nodes during reoperation, many methods were reported to be used for localization, such as hook-needle, intraoperative ultrasound, radio-guided surgery, and dyes.^[20–22]^ With respect to the dyes, the initial lymph tracer was methylene blue, which may cause local infection and it diffused so easily that the time of surgery was limited.^[23]^ Afterward, charcoal tattooing was extensively used as lymph tracer for tumor operation, but it lacks the directivity for lymphatic system because of its varied particle size. However, due to the highly uniform size in diameter, CNs effectively solve this problem. Mathieu et al^[24]^ have indicated that the carbon black material is not easy to be dissipated in lymph nodes with a duration of 6 months after dyeing without any inflammatory reaction in the dyeing area (no white blood cells and lymphocyte infiltration). van Tongeren et al^[25]^ showed that the raw material of CNs was carbon black and it didn’t cause mutagenicity or carcinogenicity in mice. Magrez et al^[26]^ showed that CNs have no significant effect on the central nervous system, cardiovascular system, and respiratory system. In our present study, we observed no side effect related to CNs injection including infection, tissue necrosis, and fever.

Commonly, the lymph tracer was used in the course of the initial operation of thyroid.^[8,12,27]^ For the reoperation of the recurrent thyroid carcinoma, some studies reported that they located the suspicious lesions with CNs injection by surgeon under direction vision.^[28–30]^ But we thought it was inapplicable, because the suspicious lesions may be invisible in the surgical field. A few studies have reported preoperative ultrasound-guided lymph tracer injection for localization of recurrent thyroid carcinoma, but the lymph tracer they used was charcoal tattooing.^[4,31–34]^ So far, to our knowledge, there have been no reports about ultrasound-guided CNs localization for reoperation of thyroid. In our study, this procedure was performed 1 to 14 days before reoperation. The dyeing effect was perfect, and no fade was found.

Ultrasound-guided CNs injection is an easy procedure and could be used in every hospital where they can perform the fine-needle aspiration biopsy. In our study, it is safe, and no occurrence of severe complications was observed. Patients who underwent this procedure did not have any side effects such as uncomfortable feelings, bleeding, and inflammation, which are in accordance with other reports.^[4,31–34]^ Just 1 patient (3.8%) had skin marking at the puncture site, which is similar to the charcoal tattooing injection as Kang et al^[31]^ reported (4%). But it can be overcome by controlling the volume of injection and giving a negative pressure during withdrawal of the needle.

With the CNs guiding, the surgeon can easily locate the suspicious lesions in reoperation and identify parathyroids or recurrent laryngeal nerves without dyeing. In our study, the results showed that the number of resected lymph nodes in the CNs group was smaller than that in the control group, but the positive rate of resected lymph nodes in total, in the central and lateral compartments in the CNs group was significantly higher than that in the control group (*P* < 0.001, *P* < 0.001, and *P* = 0.041). Also, the CNs dyeing helps the protection of parathyroid and recurrent laryngeal nerves. In the CNs group, the rate of temporary HPT was significantly lower compared with the control group (*P* = 0.021). Moreover, there were no significant differences for permanent HPT and vocal cord paralysis between the 2 groups. All these benefits increase the safety and effectiveness of reoperation of PTC and save the operation time.

In previous studies, we know that CNs could dye the surrounding lymph nodes through the lymph vessels when the puncture site is thyroid gland in the initial operation.^[8]^ In the reoperation, we found that some lymph nodes surrounding the injected suspicious lymph node were also dyed in the surgical field. This indicated that the local lymph vessels were still unimpeded after the initial surgery. Several studies about the technology of preoperative ultrasound-guided charcoal tattooing localization showed that their reoperation methods were just the resection of the preoperative suspicious lymph nodes with dyes injection.^[4,32]^ In our study, all the patients underwent MRND or selective neck node dissection. The postoperative pathological reports showed that 89 of 426 lesions without CNs injection (20.9%) were also metastatic lymph nodes. This result signified that it is necessary to perform the dissection of all the lymph nodes in the surgical field. The reason is that the effectiveness of the existing preoperative localization depends on the sensitivity of the identification ability of ultrasound.

Whether the tracer injected into lymph node would influence the pathological examination is disputable. Soprani et al^[32]^ considered that the tracer may affect the postoperative pathological identification and should avoid injecting inside the lesion. However, in our study, pathologists did find this problem and thought that it did not influence identification. In the present study, we found that the optimal volume of CNs injection was less than 0.1 mL. Otherwise, it may cause wide diffusion. In addition, this procedure is fit for any lesion that the ultrasound could identify. In our study, the sizes of injected lymph nodes were from 5 to 19 mm. For this procedure, the experience of radiologists is important, particularly when lesions were located near the large cervical vessels. All 3 failed injected lesions occurred in the early stage of our study, when radiologists in our hospital had not been familiar with this procedure yet.

Our study had some limitations. First of all, our study was not a prospective randomized control one. Second, the number of patients in our study was small. Third, our follow-up period was just 6 months, which is inadequate for the observation of recurrence. More large randomized control prospective studies are warranted in the future.

Preoperative ultrasound-guided CNs injection is a safe and effective method for localization of the metastatic lymph nodes during reoperation. The procedure is easy to implement and inexpensive. It is useful in the resection of the metastatic lymph nodes and reduces the risk of damage toward recurrent laryngeal nerves and parathyroid glands in reoperation. It will be a good prospect for the application of preoperative ultrasound-guided CNs injection in the reoperation of thyroid cancer.

## Acknowledgments

The investigators thank the patients for their participation.
